# Multiple transluminal gateway technique for drainage of an infected walled-off pancreatic necrosis and pancreatic pseudocyst

**DOI:** 10.1055/a-2701-5119

**Published:** 2025-09-26

**Authors:** Ahmed Altonbary, Ahmed Gaheen, Asmaa Gameel, Hazem Hakim

**Affiliations:** 1 68780 Department of Gastroenterology and Hepatology, Mansoura University, Mansoura, Egypt

The multiple transluminal gateway technique (MTGT) was first described in 2011 for better drainage of walled-off pancreatic necrosis (WON) by creating multiple transluminal fistulas 1 . Three retrospective case series reported better clinical success for MTGT compared to single-access endoscopic drainage for WON 2 . The ESGE recommends the MTGT for patients with either multiple or large (>12 cm) WON, or in case of suboptimal response to the single gateway technique 2 . However, the MTGT was not frequently described in the literature for the same-session drainage of multiple peripancreatic fluid collections. Herein, we report successful same-session MTGT for drainage of an infected WON and pancreatic pseudocyst.

A 73-year male presented to our hospital with abdominal pain and fever. He was previously admitted to another hospital with biliary acute pancreatitis and discharged after a laparoscopic cholecystectomy. Laboratory investigations showed elevated C-reactive protein and total leucocytic count. Abdominal computed tomography (CT) showed a large pancreatic body WON (11 cm × 8 cm) with thick debris inside and another pancreatic tail clear cyst (8 cm × 8 cm). EUS-guided same-session MTGT was performed for the infected WON using a hot Lumen apposing metal stent (LAMS) (16 mm diameter, 31 mm flare, and 2 cm length) and for the pseudocyst using a double pigtail plastic stent (7 cm, 10 Fr) ( Fig. 1 , Video 1 ). After endoscopic management, the high fever resolved, CRP levels significantly decreased, and the patient was discharged after a few days. A follow-up CT after 2 weeks showed a significant reduction in both cyst sizes with necrotic debris inside the WON. Subsequently, gastroscopy was inserted through LAMS into the cyst cavity and all necrotic debris was removed, followed by removal of both stents ( Fig. 2 , Video 1 ). No procedure-related adverse events were reported. A follow-up CT after 6 weeks showed a residual cyst cavity, and the patient remained symptoms free.

**Fig. 1:**
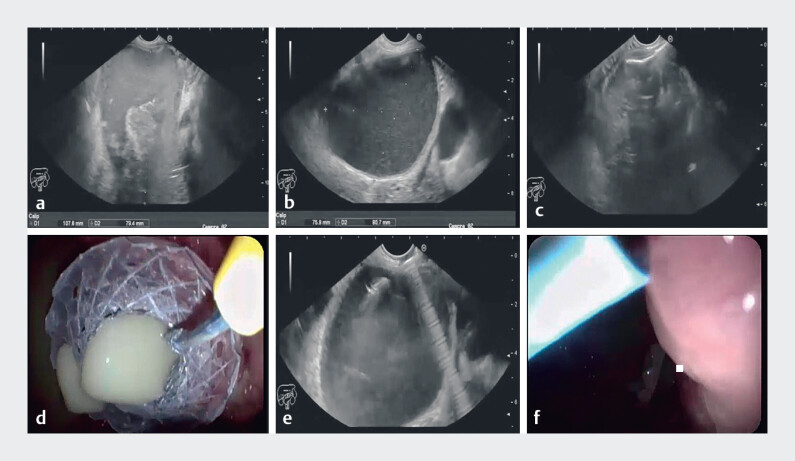
EUS-guided MTGT for infected WON and pancreatic pseudocyst: **a** WON measuring 11 cm × 8 cm with necrotic debris, **b** pancreatic tail pseudocyst measuring 8 cm × 8 cm, **c** deployment of distal flare of LAMS under EUS guidance, **d** fully deployed LAMS draining pus, **e** EUS-guided puncture of the pancreatic tail pseudocyst with 19-G needle, and **f** deployed double pigtail plastic stent and LAMS.

**Fig. 2:**
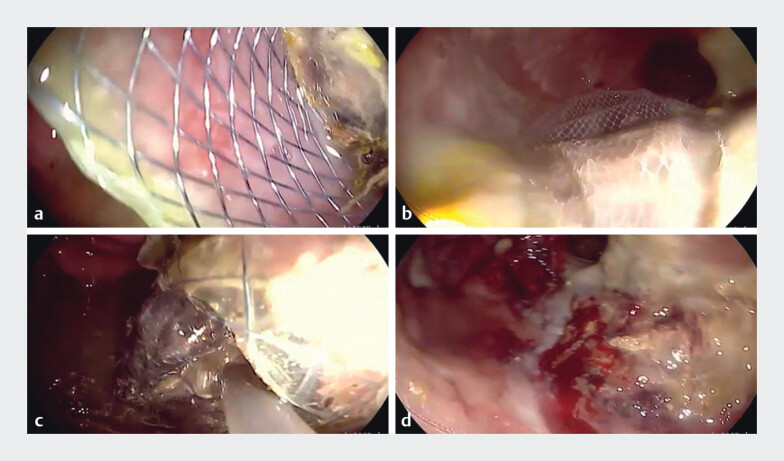
Endoscopic necrosectomy of the WON after 2 weeks: **a** necrotic debris blocking the stent, **b** removing the necrotic debris with Roth net, **c** necrotic debris removed to the gastric lumen, and **d** clean cyst cavity.

Multiple transluminal gateway technique for drainage of an infected walled-off pancreatic necrosis and pancreatic pseudocyst.Video 1

Endoscopy_UCTN_Code_TTT_1AR_2AI

## Notes

Endoscopy E-Videos https://eref.thieme.de/e-videos
*E-Videos* is an open access online section of the journal *Endoscopy* , reporting on interesting cases and new techniques in gastroenterological endoscopy. All papers include a high-quality video and are published with a Creative Commons CC-BY license. Endoscopy E-Videos qualify for HINARI discounts and waivers and eligibility is automatically checked during the submission process. We grant 100% waivers to articles whose corresponding authors are based in Group A countries and 50% waivers to those who are based in Group B countries as classified by Research4Life (see: https://www.research4life.org/access/eligibility/ ). This section has its own submission website at https://mc.manuscriptcentral.com/e-videos .
